# Supplementary material for: Dominant role of CDKN2B/p15INK4B of 9p21.3 tumor suppressor hub in inhibition of cell-cycle and glycolysis
Source: Nat Commun. 2021 Apr 6;12:2047. doi: 10.1038/s41467-021-22327-5 (PMC8024281; doi:10.1038/s41467-021-22327-5)
Supplement: Supplementary file 1 — Supplementary Information [file 41467_2021_22327_MOESM1_ESM.pdf]

Supplementary Table 1. Proteins bound to p15 in pull-down essay as identified by mass spectrometry

LC-MS/MS Protein Identified	Mascot Score	# Peptide sequences Identified	NCBI or SwissProt Accession #	MW
#1				
GroEL [Escherichia coli]	915	29	gi|18028150	57446
#2				
Tubulin, beta [Homo sapiens]	31	4	gi|18088719	50208
#3				
succinate dehydrogenase flavoprotein subunit [Escherichia coli]	2329	92	gi|446698195	65180
T-complex protein 1 subunit gamma isoform c [Homo sapiens]	220	9	gi|58761484	57035
#4				
alpha-enolase isoform 1 [Homo sapiens]	1823	117	gi|4503571	47566
#5				
enolase [Homo sapiens]	138	6	gi|4503571	47566
#6				
imidazoleglycerol-phosphate dehydratase [Escherichia coli]	153	4	gi|446002283	40724
succinate dehydrogenase flavoprotein subunit [Escherichia coli]	71	1	gi|446698195	65180
#7				
heterogeneous nuclear ribonucleoprotein H1 (H), isoform CRA_b	105	4	gi|5031753	44109
#8				
bifunctional chorismate mutase/prephenate dehydratase [Escherichia coli]	40	1	gi|446122246	43456
IEKIIVNLISNALKHTPVHGK, 775.82(3+)				
#9				
Chain A, Crystal Structure Of Ahpc With Active Site Cysteine Mutated To Serine (C46s)	96	4	gi|30749577	20658
#10				
Keratin 10 [Homo sapiens]	27	5		63592
#11				
Keratin 1 [Homo sapiens]	45	17	gi|11935049	66240
#12				
Keratin 8	55	2	gi|181573	53529
Keratin 4	55	3	gi|38014092	35778
#13				
Chain B, E. Coli 2-Oxoglutarate Dehydrogenase (E1o)	1688	74	gi|134104926	105720
lysyl hydroxylase isoform 2 [Homo sapiens]	34	2	gi|2138314	85533
procollagen-lysine, 2-oxoglutarate 5-dioxygenase 2 isoform b variant [Homo sapiens]	34	2	gi|62089344	91844
#14				
BNIPXL-beta [Homo sapiens]	39	1	gi|38259615	82094
KIAA0367, isoform CRA_c [Homo sapiens]	39	1	gi|119582987	86718
#15				
protein-tyrosine kinase (EC 2.7.1.112), receptor type tie precursor - human	24	3	gi|107565	127746
#16				
spermidine N1-acetyltransferase [Escherichia coli]	77	5	gi|447055050	22042
#17				
gluconate kinase 2; gluconate transport, GNT I system [Escherichia coli]	150	3	CUX82475.1	19479

Supplementary Table 2. Oligonucleotide primers used in this study

Application	Designation	Primer sequence
Genotyping	UpII-HRas	forward:5'-TCCCACTCCGAGACAAAATC-3'<br>reverse: 5'-ATTCGTCCACGAAGTGGTTC-3'
	p15 KO	forward: 5'-ATCCGAGTGCCTACACCTCCA-3'<br>reverse: 5'-GCTCCCGATTCGCAGCGCAT-3'
	p15 WT	forward: 5'-GTCATGATGATGGGCAGCG-3'<br>reverse: 5'-CCGGAATTCGCGTGCAGATACCTCGC-3'
	p16 KO	forward: 5'-GACTCCATGCTGCTCCAGAT-3'<br>reverse: 5'-GCCGCTGGACCTAATAACTTC-3'
	p16 WT	forward: 5'-GGCAAATAGCGCCACCTAT-3'<br>reverse: 5'-GACTCCATGCTGCTCCAGAT-3'
	Ink4ab KO	Forward: 5'-GCAGTGTTGCAGTTTGAACCC-3'<br>Reverse: 5'-TGTGGCAACTGATTCAGTTGG-3'
Myc-tagging	p15	forward: 5'-TCAGCTAGCATGCGCGAGGAGAACAAGG-3'<br>reverse: 5'-ACGATCGATTCACAGATCCTCTTCTGAGATGAGTTTCTGCTCG TCCCCCGTGGCTGTGCG-3'
	p16	forward : 5'-AAAGCTAGCATGGAGCCG GCG GCG GGG A-3'<br>reverse: 5'-ACGATCGATTCACAGATCCTCTTCTGAGATGAGTTTCTGCTCA TCGGGGATGTCTGAGGG-3'
Domain switch between p15 and p16	primer 1	5'-TCAGCTAGCATGCGCGAGGAGAACAAGGGC-3'
	primer 2	5'-TCATGACCTGGATCGCGCGCCT-3'
	primer 3	5'-GCGCGCGATCCAGGTCATGATGATGGGC-3'
	Primer 4	5'- ACGATCGATTCAGATATCATTTGCTGCCAGATCCT-3'
	primer 5	5'-AAAGCTAGCATGGAGCCGGCGGCGGGGA-3'
	primer 6	5'-TGCAGCACCACCAGCGTGT-3'
	primer 7	5'-TCCTGGACACGCTGGTGGT-3'
Histidine tagging	p15	forward: 5'-AGAACCATGGATGCGCGAGGAGAACAAGGGC-3'<br>reverse: 5'-AATACTCGAGGTCCCCCGTGGCTGTGCGCA-3'
	p16	forward: 5'-AGAACCATGGATGGAGCCGGCGGCGGGGA-3'<br>reverse: 5'-AATACTCGAGATCGGGGATGTCTGAGGGACCTT-3'
p15 N-terminal deletion	Δ2-15	forward: 5'-GATGAGGGTCTGGCCAGC-3'
	Δ2-27	forward: 5'-GAGAAGGTGCGACAGCTCCT-3'
	Δ2-37	forward: 5'-GCGGATCCCAACGGAGTCA-3'
	Δ2-49	forward: 5'-GCGATCCAGGTCATGATGATG-3'
	Reverse	reverse: 5'-CATGCTAGCACGCGTCAGCT-3'
p15 mutations	E16A	forward: 5'-GATGCGGGTCTGGCCAGCG-3'<br>reverse: 5'- GCTGCCGCCCCCACTGG-3'
	S20A	forward: 5'-GCGGCGCGGGGACTAGTGG-3'<br>reverse: 5'-GGCGGCGGCCAGACCCTC-3'
	R24A	forward: 5'-GCGGCGGGACTAGTGGAGAAG-3'<br>reverse: 5'-CGCGGCGCTGGCCAGACC-3'
	R45A	forward: 5'-GCTTTCGGGAGGCGCGCGATC-3'<br>reverse: 5'-GTTGACTCCGTTGGGATCCGC-3'
p16 mutation	R24L	forward: 5'- CTGGTAGAGGAGGTGCGGG-3'<br>reverse: 5'-ACCCCGGGCCGCGGCC-3'

Supplementary Table 3. Total simulation time for molecular dynamics simulation

System	Atoms	Box size	Ions	Time for each replica	Num of replicas
p16-CDK6 complex	71,906	85 × 98 × 103	2 Na^+	150 ns	5
p15-CDK6 complex	66,357	86 × 88 × 105	2 Na^+	150 ns	5
$p16^{R24L}$-CDK6 complex	71,899	85 × 98 × 103	3 Na^+	150 ns	5

Supplementary Table 4. Antibodies used in this study

Antibody name	Source	Catalog #	Method	Dilution	Supplier
anti-Up3a	mouse	N/A	IHC/IF*	1:300	In-house made
anti-Keratin 5	rabbit	ab52635	IHC/IF	1:200	Abcam
anti-Keratin 14	mouse	ab7800	IHC/IF	1:500	Abcam
anti-Ki67	rabbit	ab15580	IHC/IF	1:250	Abcam
anti-Myc-tag	mouse	#2276	IP	1:1000	Cell Signaling Technology
anti-CDK4	rabbit	#12790	WB	1:1000	Cell Signaling Technology
anti-histidine-tag	rabbit	#12698	WB	1:1000	Cell Signaling Technology
anti-CDK6	rabbit	#13331	WB	1:1000	Cell Signaling Technology
anti-ENO1	chicken	E3222-75	WB	1:500	United States Biological
anti-ENO2	rabbit	ab53025	WB	1:1000	Abcam
anti-p15	rabbit	ab53034	WB	1:1000	Abcam
Anti-p19	mouse	Sc-1665	WB	1:500	Santa Cruz Biotechnology
anti-PKM1	rabbit	#7067S	WB	1:1000	Cell Signaling Technology
anti-p16	rabbit	ab51243	WB	1:1000	Abcam
anti-phos-ERK1/2	rabbit	#4370S	WB	1:1000	Cell Signaling Technology
anti-ERK1/2	rabbit	#4695S	WB	1:1000	Cell Signaling Technology
anti-phos-RB1 Ser780	rabbit	ab47763	WB	1:1000	Abcam
anti-phos-RB1 Ser801/811	rabbit	#8516S	WB	1:1000	Cell Signaling Technology
anti-pan-RB1	rabbit	#9313S	WB	1:1000	Cell Signaling Technology
anti-phos-p130 Ser 952	rabbit	ab68136	WB	1:50000	Abcam
anti-phos-p107 Ser 975	rabbit	sc-130209	WB	1:800	Santa Cruz Biotechnology
anti-β-Actin	mouse	A1978	WB	1:5000	Sigma

* Abbreviations: IHC, immunohistochemistry; IF, immunofluorescence; IP, immunoprecipitation; WB, Western blotting.

Supplementary Fig. 1

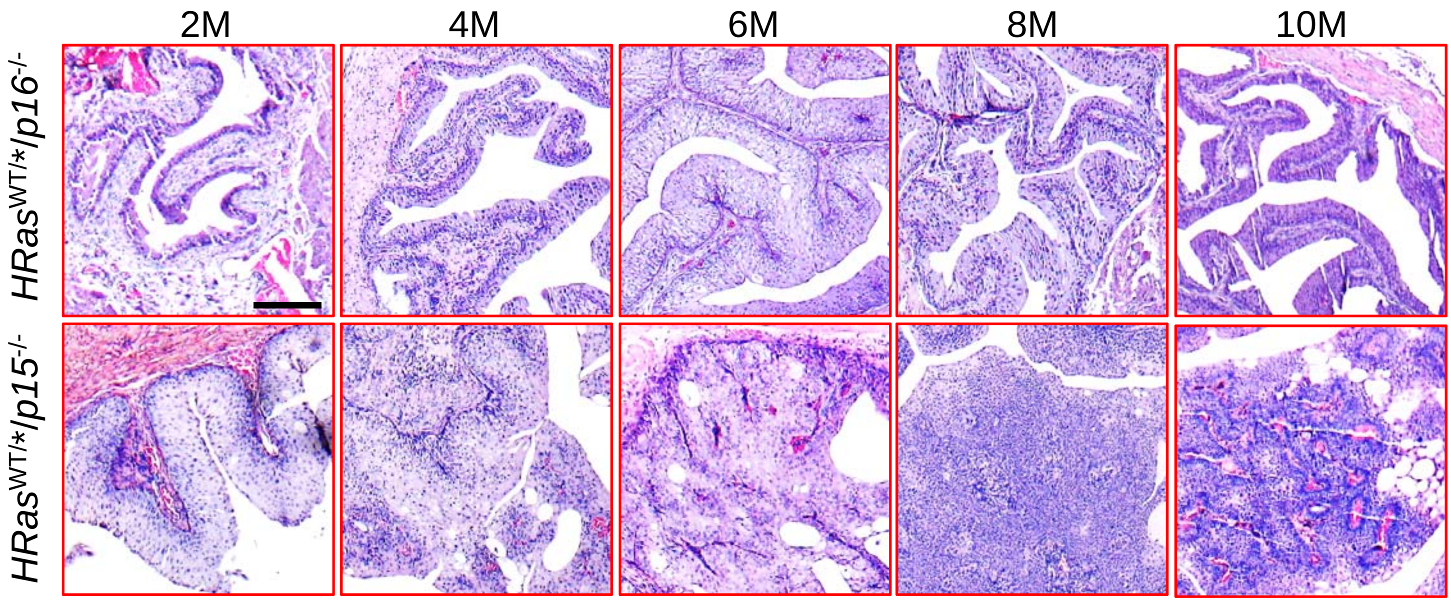

Supplementary Figure 1. High magnification of the red-boxed areas in Fig. 2e, illustrating that *HRas*$^{WT/*}$/*p16*$^{-/-}$ mice only had hyperplastic urothelial folds and were devoid of any bladder tumor throughout the observation period, and that the *HRas*$^{WT/*}$/*p15*$^{-/-}$ mice developed urothelial hyperplasia at 2 months of age and superficial bladder tumors that were of low pathological grade and non-invasive from 4-10 months of age. All panels are the same magnification and the scale bar in the upper-left panel equals to 100 μm. n = 10 mice per genotype per time point.

Supplementary Fig. 2

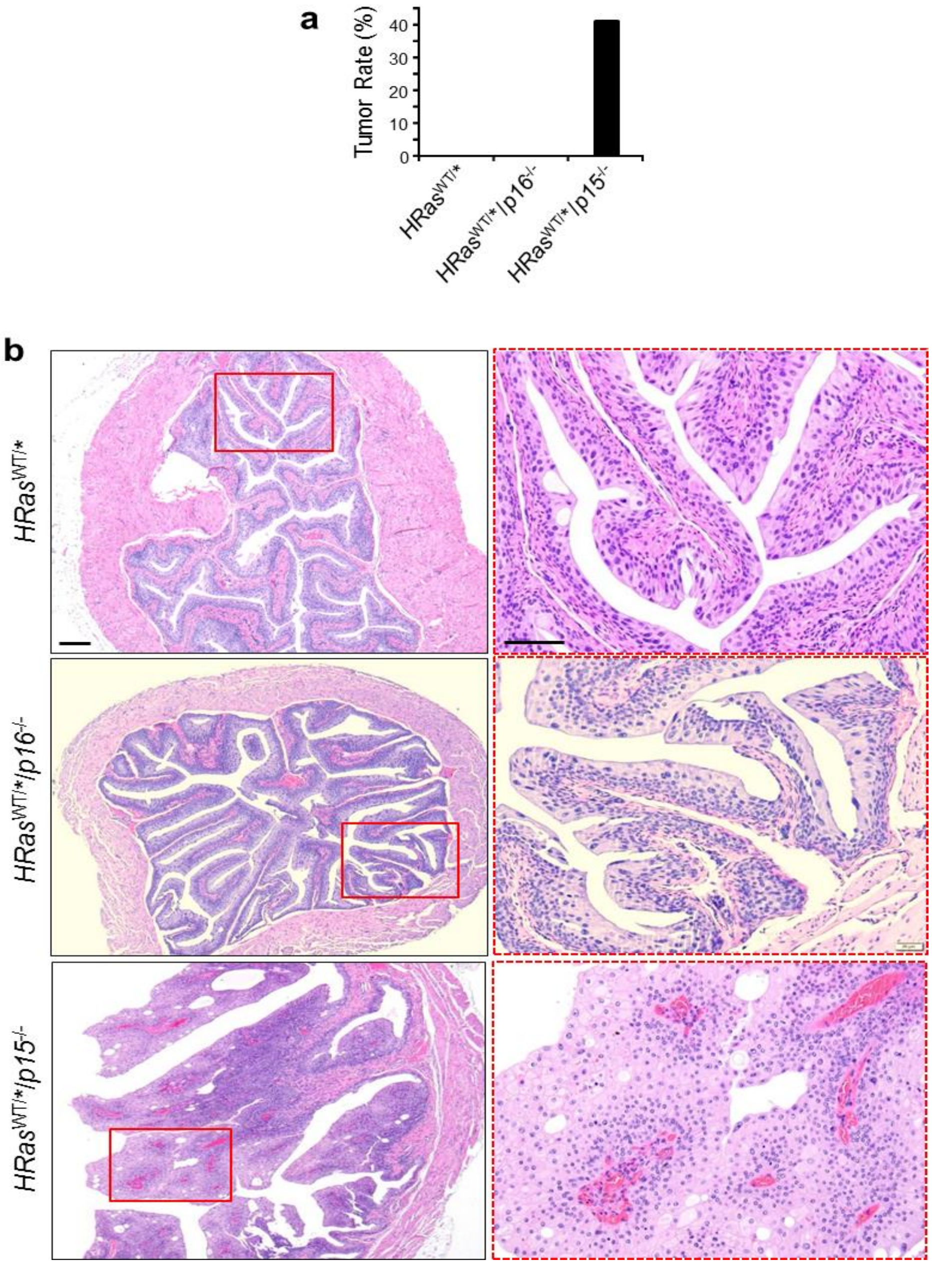

Supplementary Figure 2. An independent cohort showing different propensity of tumorigenesis in urothelial cells expressing *HRas** and lacking p15 versus p16. (a) Three groups of mice (all at 3 months of age) were compared: (i) *HRas*$^{WT/*}$ (n=10); (ii) *HRas*$^{WT/*}$/*p16*$^{-/-}$ (n=10) and (iii) *HRas*$^{WT/*}$/*p15*$^{-/-}$ (n=15). Tumor rate was expressed as % of mice that developed urothelial tumors. Note that 45% of the *HRas*$^{WT/*}$/*p15*$^{-/-}$mice but none of *HRas*$^{WT/*}$/*p16*$^{-/-}$ mice had bladder tumors. (b) Representative images of the three genotypes shown in (a). Note that *HRas*$^{WT/*}$ and *HRas*$^{WT/*}$/*p16*$^{-/-}$mice exhibited mucosal folds in contracted bladders (left panels) that were hyperplastic but devoid of any tumor (right panels showing higher magnification of the boxed areas in the left panels), whereas *HRas*$^{WT/*}$/*p15*$^{-/-}$mice contained multifocal urothelial tumors that were comprised of complex branching structures and fibrovascular cores. Bar in upper left panel represents all the left panels and equals to 200 μm; that in upper right panel represents all the right panels and equals to 50 μm.

Supplementary Fig. 3

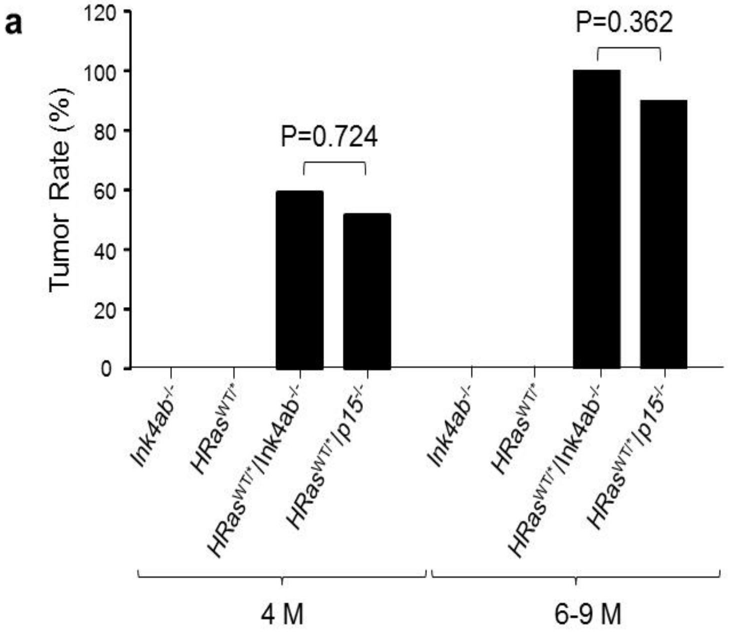

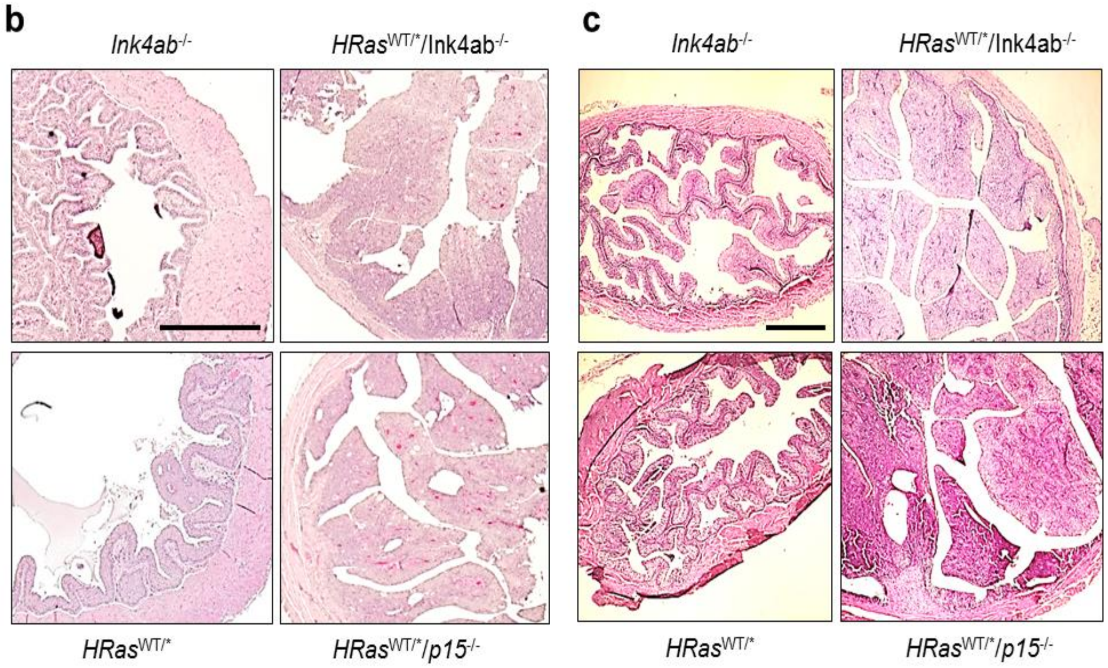

Supplementary Figure 3. Loss of both p15 and p16 did not significantly enhance urothelial tumorigenesis in urothelial cells expressing *HRas* beyond loss of p15 alone. (a) Two separate cohorts of mice with genotypes indicated below each column (the first cohort of mice all at 4 months of age; n=10, 10, 15 and 15; and the second cohort of mice between 6-9 months of age; n=6, 6, 10 and 10). Note that, while the tumor rate was slightly higher in *HRas*$^{WT/*}$/Ink4ab$^{-/-}$ mice than in *HRas*$^{WT/*}$/p15$^{-/-}$ mice, it did not have statistical significance. (b and c) Representative H&E images of the bladders from mice from the first (b) and second cohort (c) showing tumors morphologically indistinguishable from those from *HRas*$^{WT/*}$/p15$^{-/-}$ mice (Fig. 1f and Fig. 2e). Scale bars in upper left panels of (b and c) equal to 500 μm. Numbers of mice analyzed for each genotype were identical to those indicated in (a).

Supplementary Fig. 4

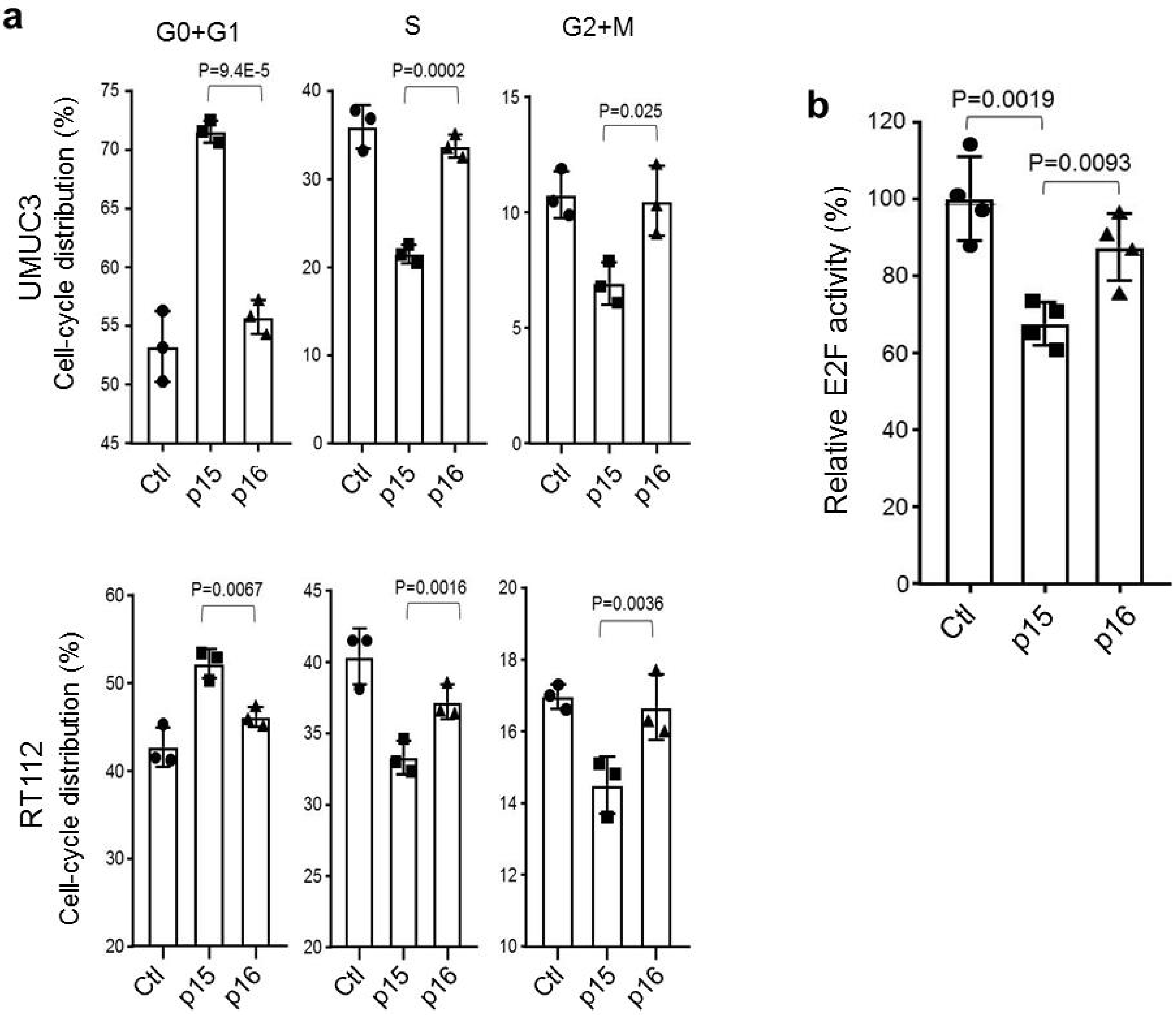

Supplementary Figure 4. Effects of enforced p15 or p16 expression on cell-cycle progression and E2F activity. (a) Human UMUC3 and RT112 cell lines were stably transfected and induced to express either p15 or p16, using a two-vector, tetracycline-inducible system. Cell-cycle analysis (FACS) was done 48 hours after the induction of protein expression. All cell experiments were done in triplicates. Note the much larger fractions of G0+G1 cells and smaller fractions of S and G2+M cells in p15 transfected cells than in p16 transfected cells. n = 3 of biologically independent samples. Data are presented as mean value +/- SD. Two-sided *t*-test was performed to compare the significance of the difference between the two groups (p15 versus p16). The P values are shown in the figure. (b) E2F activity, as evidenced by a reporter assay (see Methods), was significantly lower in p15 transfected UMUC3 cells than in p16 transfected UMUC3 cells. n = 4 of biologically independent samples. Data are presented as mean values +/- SD. Two-sided *t*-test was performed to compare the significance of the difference between every two groups. The P values are shown in the figure.

Supplementary Fig. 5

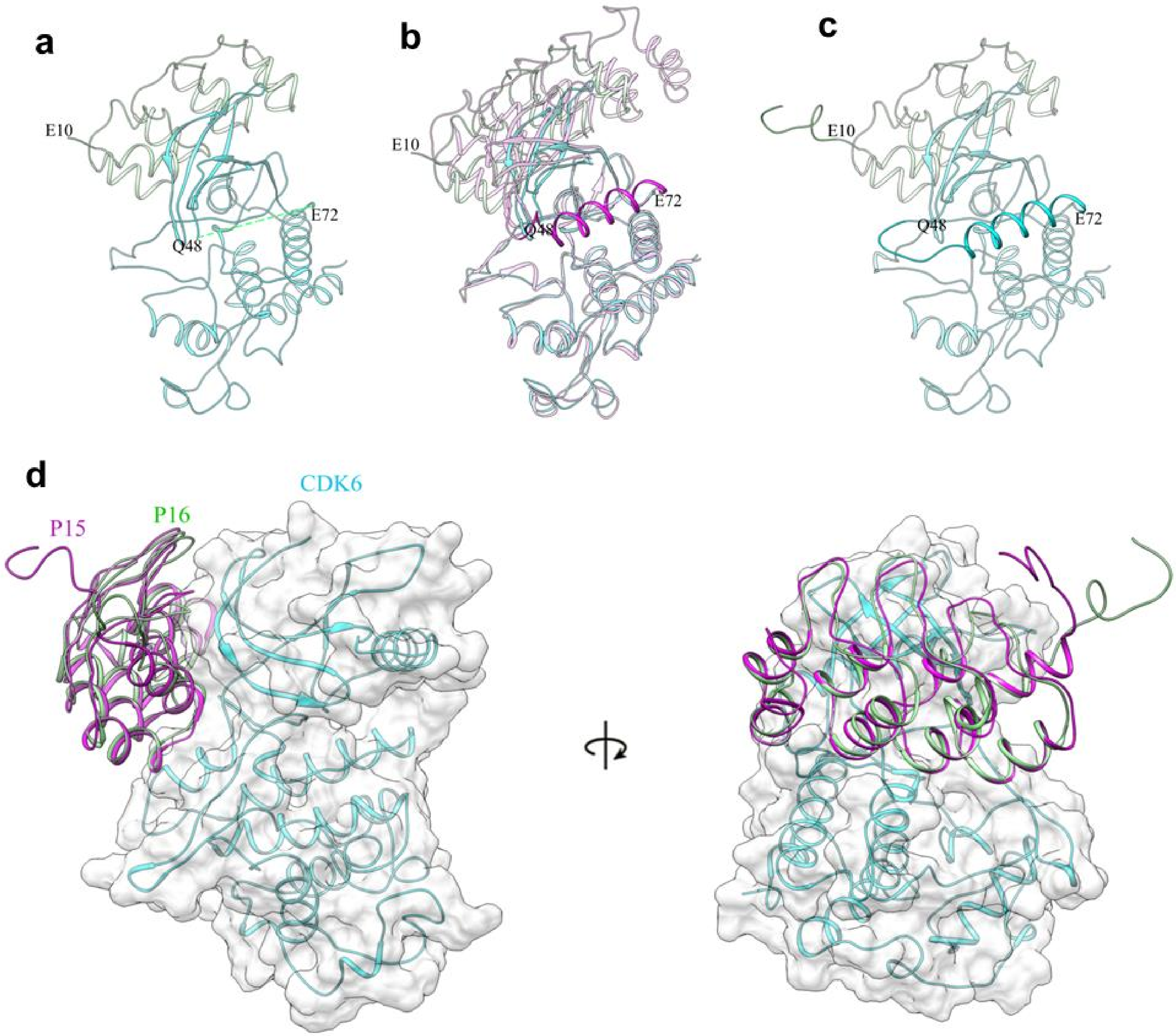

Supplementary Figure 5. Computational modeling of the missing parts of p16-CDK6 complex (PDB 1bi7). (a) p16-CDK6 complex (PDB id: 1bi7) fetched from PDB database. p16 and CDK6 are colored light green and cyan, respectively. Residues connecting with the missing parts are labelled. (b) p18-CDK6-Kcyclin complex (PDB id: 1g3n) is superimposed on the p16-CDK6 complex. The missing part (sequence 49-71) of CDK6 is built on the basis of the CDK6 structure (colored magenta) from p18-CDK6-Kcyclin complex. (c) A complete p16-CDK6 complex is built, in which the two missing parts are modeled by using Modeller program. (d) Computational modeling of p15-CDK6 complex on the basis of p16-CDK6.

Supplementary Fig. 6

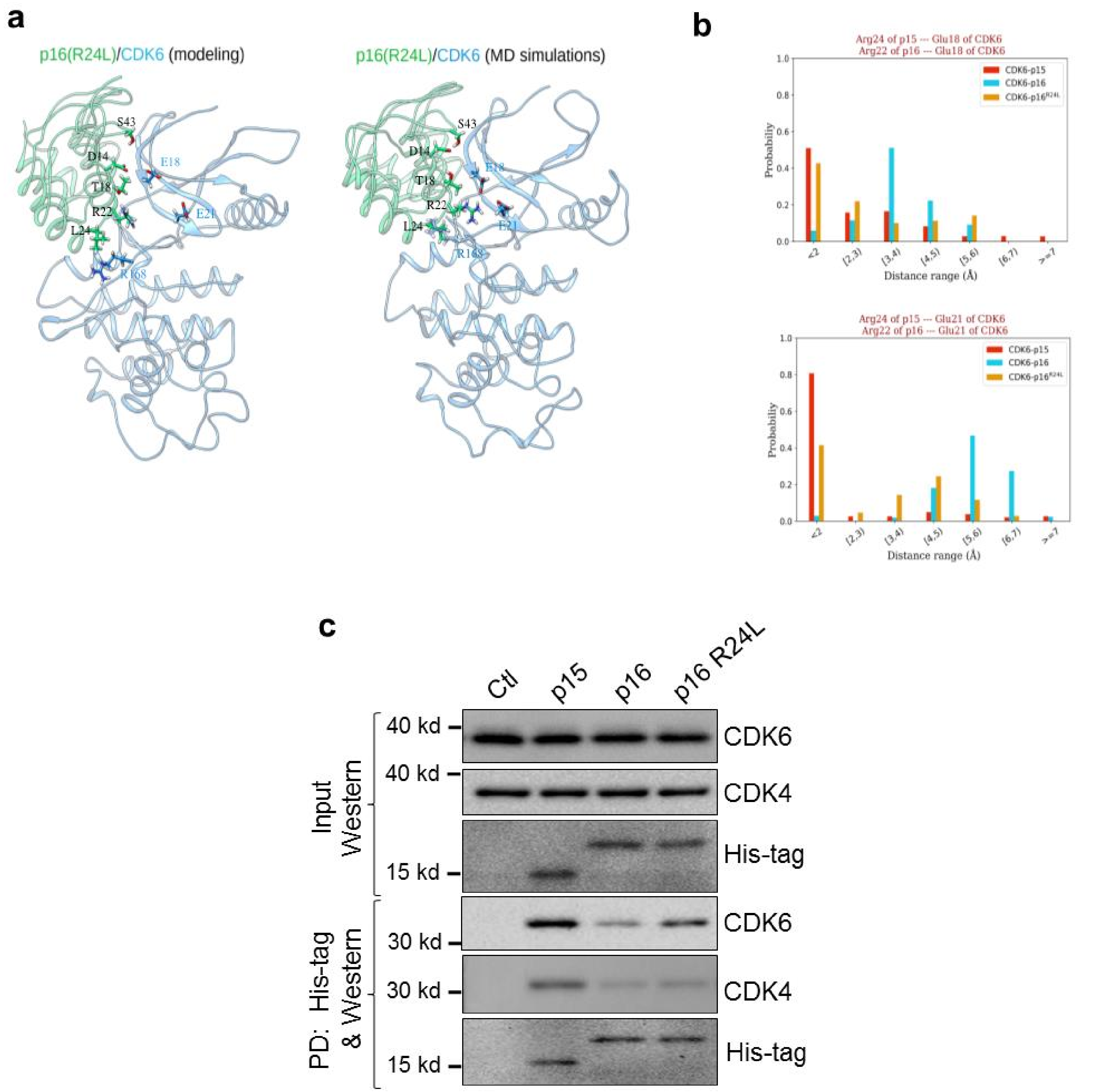

Supplementary Figure 6. Molecular dynamics simulations of p16^{R24L}-CDK6 complex and experimental validation. (a) To further test the hypothesis that the relatively weak interaction between p16 and CDK6 was due in part to the repulsive force between Arg24 of p16 (corresponding to Leu26 of p15) and Arg168 of CDK6, Arg24 of p16 was mutated to leucine in starting configuration (left panel) and one representative structure in MD simulations (right panel) showed salt-bridge formation between Arg22 of p16 and Glu18 of CDK6. (b) Minimum distance distribution analysis of structures over MD simulations showed that after the R24L mutation of p16, Arg22 of p16 can partially recover the salt-bridges with Glu18 and Glu21 of CDK6. (c) Site-directed mutagenesis. Arg24 in p16 was replaced with leucine and the mutated, histidine-tagged p16 was expressed in E. coli, immobilized to nickel column and incubated with total protein extracts from UMUC3 cell line and the bound proteins were subjected to immunoblotting using antibodies against CDK4, CDK6 and histidine. Note the increased binding of mutated p16 to CDK6, but not to CDK4 (see Results for details). The experiments were repeated three times with similar results. Source data are provided as a Source Data file.

Supplementary Fig. 7

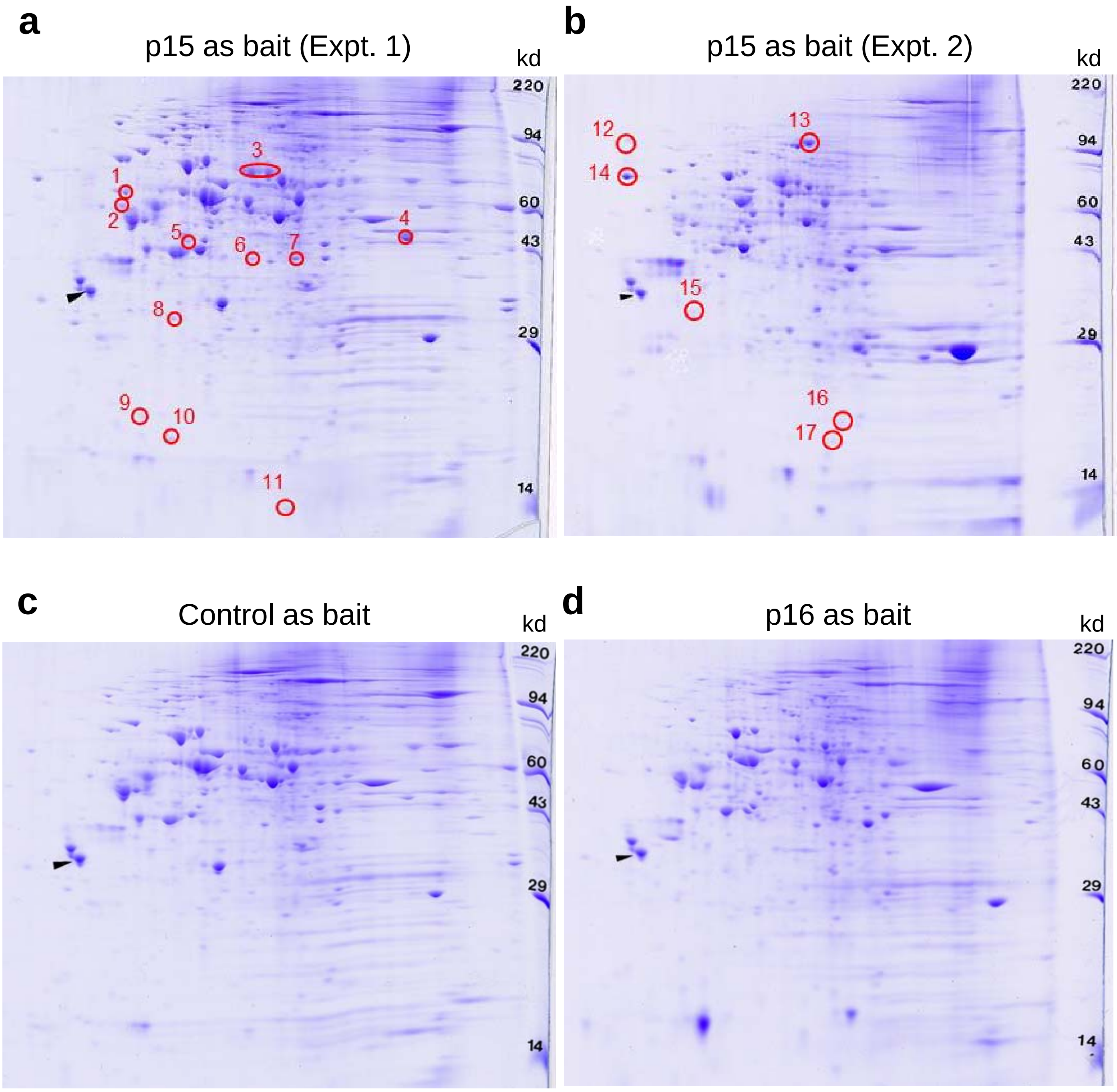

Supplementary Figure 7. (a-d) Identification of p15-binding proteins. Proteins were pulled down from nickel columns immobilized with E. coli-produced protein baits: histidine only (control, (c)), histidine-tagged p16 (d) or histidine-tagged p15 (two independent experiments, (a and (b)), and were then resolved separately by two-dimensional gel electrophoresis (the first dimension, isoelectric focusing, pH3-10; the second dimension, 10% SDS-PAGE) followed by Coomassie Blue staining. Protein spots present in the p15 group, but absent in the control group and absent or weaker in the p16 group were selected for tryptic digestion, HPLC and mass spectrometry.

Supplementary Fig. 8

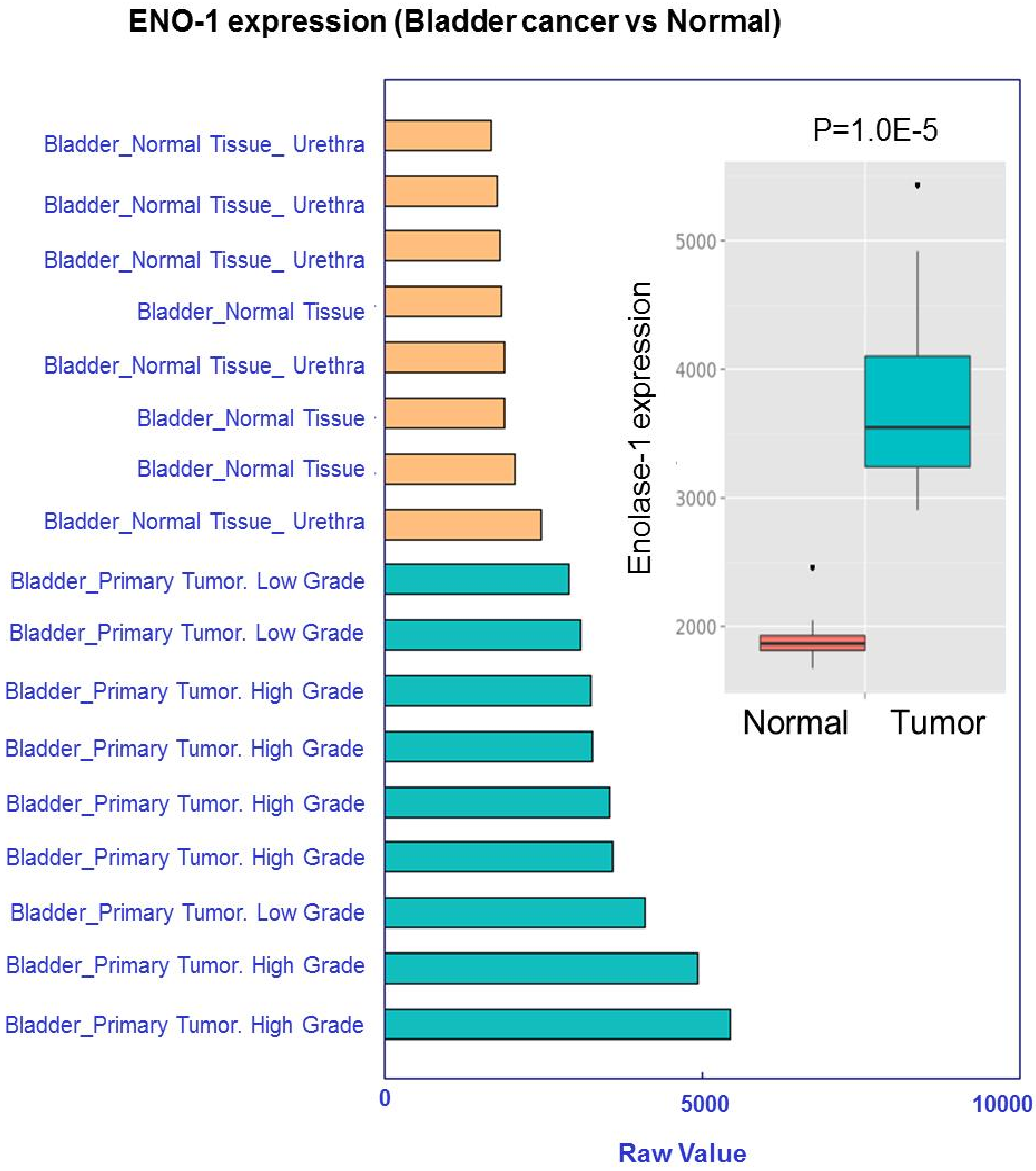

Supplementary Figure 8. Enolase-1 expression in human bladder cancer versus normal urothelial tissues. All the bladder cohorts available at the time of analysis from the cBioPortal for Cancer Genomics were assessed. Note the marked overexpression of enolase-1 in the tumor tissues (n = 9) compared to the normal tissues (n = 8). Data are presented as box plots with minima, maxima, center, 25th percentile and 75th percentile. Two-sided *t*-test was performed to compare the significance of the difference between the two groups (tumor tissue vs normal tissue). The p value is shown in the figure.

Supplementary Fig. 9

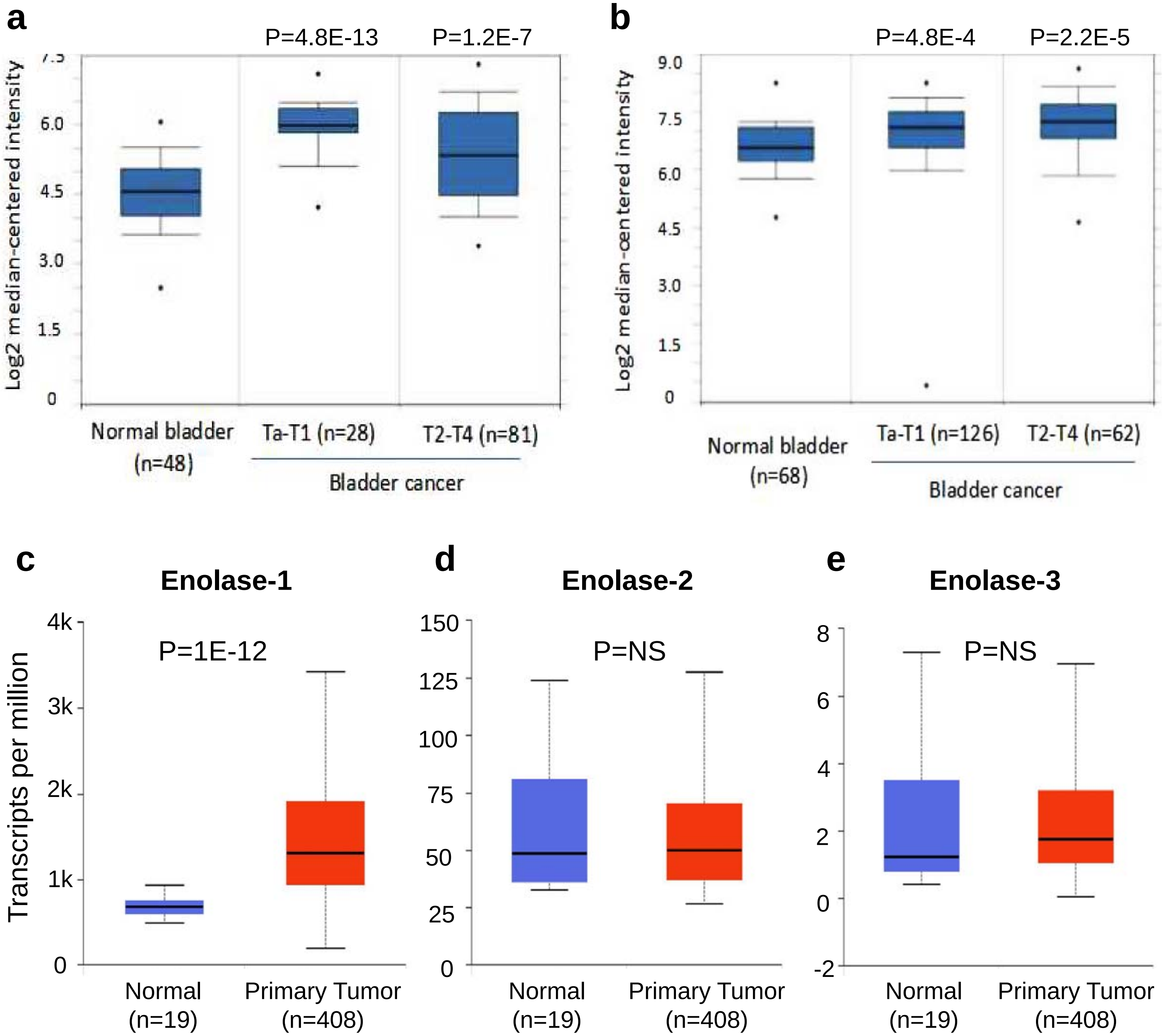

Supplementary Figure 9. Enolase-1 expression in human bladder cancer in relation to tumor stages. Cohort (a) [1] and Cohort (b) [2] both showed significantly higher enolase-1 expression in tumor tissues than in normal controls. However, there was a lack of apparent stage association of enolase-1 expression. (c-e) Expression of enolase isoforms in human bladder cancer (TCGA cohort; Ref. 3). Of the three enolase isoforms, enolase-1 is the dominant form in tumor tissues that is overexpressed in primary bladder cancer compared to normal controls and is over 20-fold higher than enolase-2 and over 500-fold higher than enolase-3. Data are presented as box plots with minima, maxima, center,10th, 25th, 75th and 90th percentile. Two-sided *t*-test was performed to compare the significance of the difference between every two groups (tumor tissue vs normal tissue). The P values are shown in the figure.

Supplementary Fig. 10

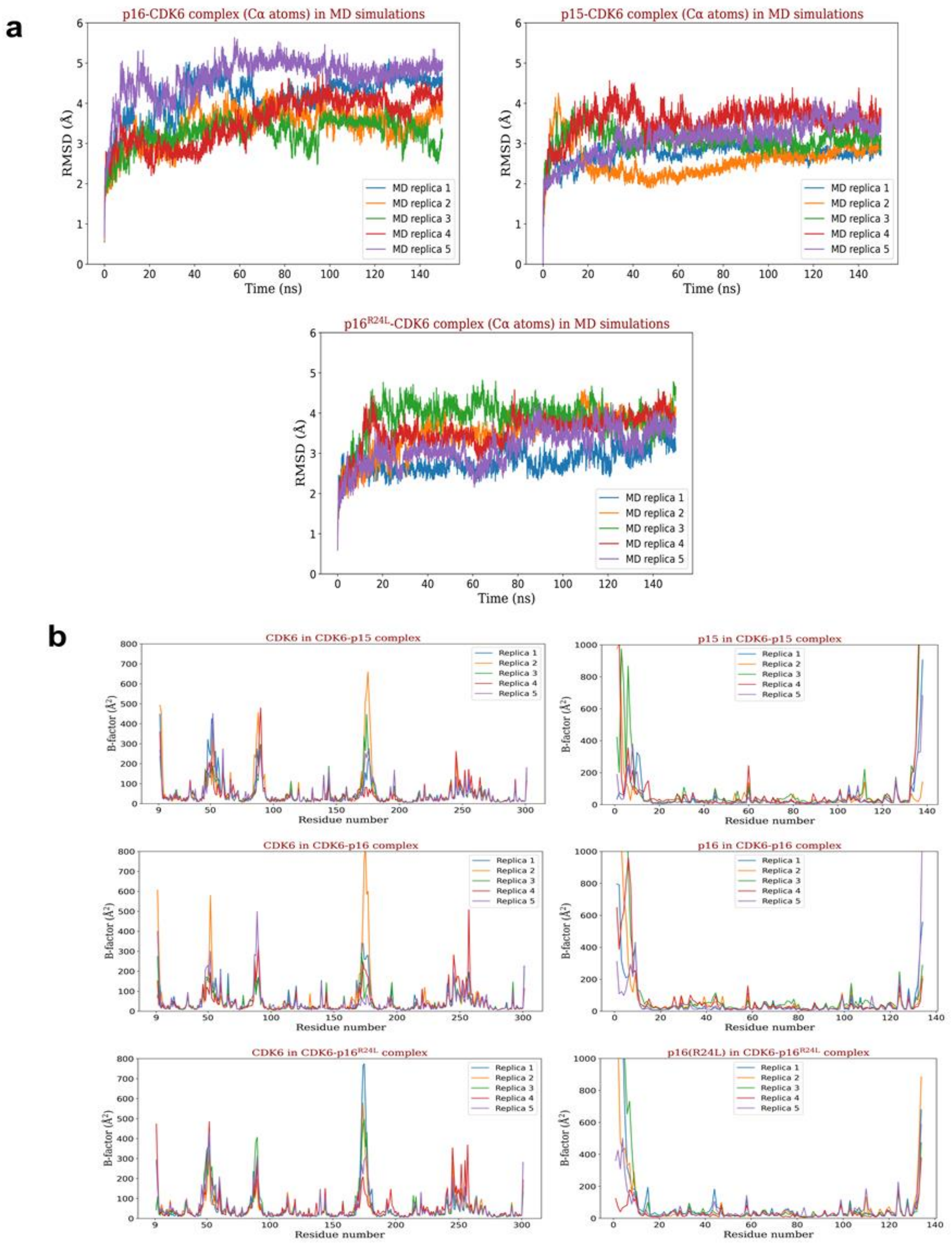

Supplementary Figure 10. (a) Protein C alpha atoms RMSD fluctuation profiles as a function of time over MD simulations of p16-CDK6, p15-CDK6 and p16^{R24L}-CDK6 complexes in all MD replicas. (b) B-factor analysis of residues of p15-CDK6, p16-CDK6 and p16^{R24L}-CDK6 complexes in all MD replicas.

Supplementary References

1. Sanchez-Carbayo M, Socci ND, Lozano J, Saint F, Cordon-Cardo C. Defining molecular profiles of poor outcome in patients with invasive bladder cancer using oligonucleotide microarrays. *J. Clin. Oncol.* **24**, 778-789 (2006).
2. Lee JS, *et al.* Expression signature of E2F1 and its associated genes predict superficial to invasive progression of bladder tumors. *J. Clin. Oncol.* **28**, 2660-2667 (2010).
3. Robertson AG, *et al.* Comprehensive Molecular Characterization of Muscle-Invasive Bladder Cancer. *Cell.* **171**, 540-556.e525 (2017).
