# Supplementary material for: Dominant role of CDKN2B/p15INK4B of 9p21.3 tumor suppressor hub in inhibition of cell-cycle and glycolysis
Source: Nat Commun. 2021 Apr 6;12:2047. doi: 10.1038/s41467-021-22327-5 (PMC8024281; doi:10.1038/s41467-021-22327-5)
Supplement: Supplementary file 3 — Reporting Summary [file 41467_2021_22327_MOESM3_ESM.pdf]

nature research

Corresponding author(s): Xue-Ru Wu

Last updated by author(s): Mar 8, 2021

Reporting Summary

Nature Research wishes to improve the reproducibility of the work that we publish. This form provides structure for consistency and transparency in reporting. For further information on Nature Research policies, see our Editorial Policies and the Editorial Policy Checklist.

Statistics

For all statistical analyses, confirm that the following items are present in the figure legend, table legend, main text, or Methods section.

n/a	Confirmed	
☐	☒	The exact sample size (n) for each experimental group/condition, given as a discrete number and unit of measurement
☐	☒	A statement on whether measurements were taken from distinct samples or whether the same sample was measured repeatedly
☐	☒	The statistical test(s) used AND whether they are one- or two-sided<br>*Only common tests should be described solely by name; describe more complex techniques in the Methods section.*
☒	☐	A description of all covariates tested
☒	☐	A description of any assumptions or corrections, such as tests of normality and adjustment for multiple comparisons
☐	☒	A full description of the statistical parameters including central tendency (e.g. means) or other basic estimates (e.g. regression coefficient) AND variation (e.g. standard deviation) or associated estimates of uncertainty (e.g. confidence intervals)
☐	☒	For null hypothesis testing, the test statistic (e.g. F, t, r) with confidence intervals, effect sizes, degrees of freedom and P value noted<br>*Give P values as exact values whenever suitable.*
☒	☐	For Bayesian analysis, information on the choice of priors and Markov chain Monte Carlo settings
☒	☐	For hierarchical and complex designs, identification of the appropriate level for tests and full reporting of outcomes
☒	☐	Estimates of effect sizes (e.g. Cohen's d, Pearson's r), indicating how they were calculated

Our web collection on statistics for biologists contains articles on many of the points above.

Software and code

Policy information about availability of computer code

Data collection	Modeler Program (v9.20)
Data analysis	ANOVA (open source); cBioPortal (open source); Modeler Program (v9.20); I-TASSER (v5.1); Amber 16 package (MD simulations; the topology and coordinate files preparation used AmberTools in Amber v16.11; and the 5 MD runs used the Amber package 16.11 version, and the 10 MD runs used the Amber package 20.06 version); SHAKE algorithm (no version information available); FlowJo (v10.4.2); ImageJ (1.52n); SPSS v17.0.

For manuscripts utilizing custom algorithms or software that are central to the research but not yet described in published literature, software must be made available to editors and reviewers. We strongly encourage code deposition in a community repository (e.g. GitHub). See the Nature Research guidelines for submitting code & software for further information.

Data

Policy information about availability of data

All manuscripts must include a data availability statement. This statement should provide the following information, where applicable:

- Accession codes, unique identifiers, or web links for publicly available datasets
- A list of figures that have associated raw data
- A description of any restrictions on data availability

All the data generated or analyzed during this study are included in this published article and its supplementary information files. The source data on ENO-1 expression in human bladder cancer cohorts shown in Supplementary Fig. 8 are available in a public repository from the https://www.cbioportal.org/ website. MD simulation data of three protein complexes are available at: https://www.nyu.edu/projects/yzhang/MD_data.

Field-specific reporting

Please select the one below that is the best fit for your research. If you are not sure, read the appropriate sections before making your selection.

☒ Life sciences ☐ Behavioural & social sciences ☐ Ecological, evolutionary & environmental sciences

For a reference copy of the document with all sections, see nature.com/documents/nr-reporting-summary-flat.pdf

Life sciences study design

All studies must disclose on these points even when the disclosure is negative.

Sample size	Sample sizes of the experimental animals were estimated based on the differences of the proportions of animals that will likely develop bladder tumors with partitioning of degrees of freedom to the effects between CDKN2B deficiency and the controls. Detectable differences in proportion of mice (12 mice/genotype or 10 mice/genotype) with tumors were predicted in a table (Fisher's Exact Test, 2 sided alpha =0.05, power = 80%; calculations from PASS 2008). All the experiments related to cultured cell lines were done in triplicate as per convention. Such experiments were also repeated for at least three times with similar results.
Data exclusions	No data were excluded from the analyses.
Replication	Compound mouse models demonstrating the critical importance of CDKN2B were performed in three independent studies. Two independent bladder cancer cell lines lacking 9p21.3 were used for CDKN2B restoration studies. All cell culture experiments were done in triplicates. For CDNK2B binding experiments, two different approaches were used (immunoprecitation and pull-down); Affinity-purification of CDKN2B-bound proteins were performed twice. All the replication experiments were successful.
Randomization	All mice for a given genotype was routinely randomized in all the experiments. All the cell culture wells were randomized into control and experimental groups.
Blinding	Pathological examination of bladder tumors was done in a double-blind manner (blinded for genotypes) by two pathologists. All other experiments were also blinded wherever applicable before data analysis.

Reporting for specific materials, systems and methods

We require information from authors about some types of materials, experimental systems and methods used in many studies. Here, indicate whether each material, system or method listed is relevant to your study. If you are not sure if a list item applies to your research, read the appropriate section before selecting a response.

Materials & experimental systems

n/a	Involved in the study	
☐	☒	Antibodies
☐	☒	Eukaryotic cell lines
☒	☐	Palaeontology and archaeology
☐	☒	Animals and other organisms
☒	☐	Human research participants
☒	☐	Clinical data
☒	☐	Dual use research of concern

Methods

n/a	Involved in the study	
☒	☐	ChIP-seq
☐	☒	Flow cytometry
☒	☐	MRI-based neuroimaging

Antibodies

Antibodies used

anti-Up3a, mouse, In-house made;
anti-Keratin 5, rabbit, ab52635, Abcam;
anti-Keratin 14, mouse, ab7800, Abcam;
anti-Ki67, rabbit, ab15580, Abcam;
anti-Myc-tag, mouse #2276, Cell Signaling Technology;
anti-CDK4, rabbit, #12790, Cell Signaling Technology;
anti-histidine-tag, rabbit, #12698, Cell Signaling Technology;
anti-CDK6, rabbit #13331, Cell Signaling Technology;
anti-ENO1, chicken E3222-75, United States Biological;
anti-ENO2, rabbit, ab53025, Abcam;
anti-p15, rabbit, ab53034; Abcam;
Anti-p19, mouse Sc-1665, Santa Cruz Biotechnology;
anti-PKM1, rabbit, #7067Sl Cell Signaling Technology;
anti-p16, rabbit, ab51243, Abcam;
anti-phos-ERK1/2, rabbit, #4370S, Cell Signaling Technology;
anti-ERK1/2, rabbit #4695S, Cell Signaling Technology;

	anti-phos-RB1, Ser780 rabbit, ab47763, Abcam;<br>anti-phos-RB1, Ser801/811 rabbit #8516S, Cell Signaling Technology;<br>anti-pan-RB1, rabbit #9313S, Cell Signaling Technology;<br>anti-phos-p130 Ser 952, rabbit ab68136, Abcam;<br>anti-phos-p107 Ser 975, rabbit sc-130209, Santa Cruz Biotechnology;<br>anti-β-Actin, mouse, A1978, Sigma.
Validation	anti-Up3a, mouse, In-house made, validated by immunohistochemistry and immunofluorescence;<br>anti-Keratin 5, rabbit, ab52635, Abcam, validated by immunohistochemistry and immunofluorescence;<br>anti-Keratin 14, mouse, ab7800, Abcam, validated by immunohistochemistry and immunofluorescence;<br>anti-Ki67, rabbit, ab15580, Abcam, validated by immunohistochemistry and immunofluorescence;<br>anti-Myc-tag, mouse #2276, Cell Signaling Technology, validated by immunoprecipitation;<br>anti-CDK4, rabbit, #12790, Cell Signaling Technology, validated by Western blotting;<br>anti-histidine-tag, rabbit, #12698, Cell Signaling Technology, validated by Western blotting;<br>anti-CDK6, rabbit #13331, Cell Signaling Technology, validated by Western blotting;<br>anti-ENO1, chicken E3222-75, United States Biological, validated by Western blotting;<br>anti-ENO2, rabbit, ab53025, Abcam, validated by Western blotting;<br>anti-p15, rabbit, ab53034; Abcam, validated by Western blotting;<br>Anti-p19, mouse Sc-1665, Santa Cruz Biotechnology, validated by Western blotting;<br>anti-PKM1, rabbit, #7067Sl Cell Signaling Technology, validated by Western blotting;<br>anti-p16, rabbit, ab51243, Abcam, validated by Western blotting;<br>anti-phos-ERK1/2, rabbit, #4370S, Cell Signaling Technology, validated by Western blotting;<br>anti-ERK1/2, rabbit #4695S, Cell Signaling Technology, validated by Western blotting;<br>anti-phos-RB1, Ser780 rabbit, ab47763, Abcam, validated by Western blotting;<br>anti-phos-RB1, Ser801/811 rabbit #8516S, Cell Signaling Technology, validated by Western blotting;<br>anti-pan-RB1, rabbit #9313S, Cell Signaling Technology, validated by Western blotting;<br>anti-phos-p130 Ser 952, rabbit ab68136, Abcam, validated by Western blotting;<br>anti-phos-p107 Ser 975, rabbit sc-130209, Santa Cruz Biotechnology, validated by Western blotting;<br>anti-β-Actin, mouse, A1978, Sigma, validated by Western blotting.

Eukaryotic cell lines

Policy information about cell lines

Cell line source(s)	RT112, UMUC3 and 293T were all from American Type Culture Collection (Manassas, VA, USA)
Authentication	The cell lines were used within 6 months after receipt from the supplier where they were authenticated by profiling the short-tandem repeats.
Mycoplasma contamination	The cell lines were tested negative for mycoplasma contamination by the supplier.
Commonly misidentified lines<br>(See ICLAC register)	No commonly misidentified cell lines were used in this study.

Animals and other organisms

Policy information about studies involving animals; ARRIVE guidelines recommended for reporting animal research

Laboratory animals	Mice, FVB/N, male and female, 0-10 months. The following strains were used: Upk2-HRas*, CDKN2B (p15) knockout, p16INK4A (p16) knockout, INK4AB (p15/p16) knockout.
Wild animals	No wild animals were involved.
Field-collected samples	This study did not involve animals collected from the field.
Ethics oversight	A study protocol was approved by the Institutional Animal Care and Use Committee (IACUC) of New York University School of Medicine.

Note that full information on the approval of the study protocol must also be provided in the manuscript.

Flow Cytometry

Plots

Confirm that:

☒ The axis labels state the marker and fluorochrome used (e.g. CD4-FITC).

☒ The axis scales are clearly visible. Include numbers along axes only for bottom left plot of group (a 'group' is an analysis of identical markers).

☒ All plots are contour plots with outliers or pseudocolor plots.

☒ A numerical value for number of cells or percentage (with statistics) is provided.

Methodology

Sample preparation	Cultured cells and mouse tissues were digested into cell suspension using trypsin and dispase, respectively.
Instrument	FACSCalibur analyzer
Software	FlowJo software (FlowJo 10.4.2)
Cell population abundance	Two steps were taken, the first removing the aggregates and debris and the second step removing the doublets. The purity was more than 90% of the starting material. The total number of cells counted was more than 20,000, with G0+G1 phase cells ranging (depending on specific experiments) from 40-75%, S phase cells ranging from 10-20%, and G2+M phase cells ranging from 15-45%.
Gating strategy	Forward and side scatter gating to remove debris, then width versus area gating to remove doublets, and then gating base on DNA content by FlowJo embedded cell-cycle analysis. Gating strategy is provided under Fig. 3b.

☐ Tick this box to confirm that a figure exemplifying the gating strategy is provided in the Supplementary Information.
